# Infrared and terahertz quantum technologies

**DOI:** 10.1515/nanoph-2024-0183

**Published:** 2024-04-23

**Authors:** Alessandro Tredicucci, Miriam S. Vitiello

**Affiliations:** Dipartimento di Fisica, Università di Pisa, Pisa, Italy; NEST, Istituto Nanoscienze – CNR, Pisa, Italy

**Keywords:** infrared, terahertz, intersubband transitions, quantum devices

In the realm of quantum technology, the exploration of novel materials and phenomena opens up promising avenues for the development of advanced devices. Particularly intriguing are the perspectives surrounding quantum devices operating across the infrared (IR) and Terahertz (THz) frequency range, which have garnered significant attention due to their potential applications in communication, sensing, and imaging. Central to this exploration is the exploitation of novel materials and the harnessing of intersubband transitions, offering an insight into the future of quantum technology [1].

Intersubband transitions, arising from quantum confinement effects in semiconductor heterostructures, represent a cornerstone of infrared and terahertz device design. These transitions involve the excitation of electrons between quantized energy levels within quantum wells or superlattices, enabling the manipulation of light–matter interactions at the nanoscale [2]. By engineering the band structure, the energy, dipole and lifetime of intersubband transitions can be controlled by design, hence allowing the creation of compact and efficient devices such as quantum cascade lasers (QCLs), detectors, saturable absorbers and optical components.

A recently very active research area is that of frequency combs, owing to their relevance in quantum metrology and sensing applications; here QCLs offer intriguing possibilities to spontaneously generate frequency combs, thanks to their intrinsic non-linearities and broad gain bandwidth [3]. Additionally, the basic physics of intersubband transitions still attracts considerable attention in the THz, particularly concerning thermal aspects [4], [5] and extension of the QCL emission frequency range [6]. In the mid-IR, on the other hand, the focus is mostly on improving QCL efficiency [7] and on applications, for instance as local oscillators in heterodyne spectroscopy [8], [9].

One of the key frontiers in IR and THz quantum devices lies in the selection and engineering of materials with tailored properties conducive to efficient operation in these spectral ranges. Traditional semiconductors based on III–V compounds like GaAs and InP have been widely adopted for mid-infrared and terahertz applications, with Sb-based heterostructures still being explored to extend the operation of intrubband devices, sources and detectors, towards shorter wavelengths [10]. In recent years though, progress in the growth of group IV (Si–Ge) heterostructures has continued steadily, and this platform now offers interesting perspectives both in the direction of fundamental quantum optics of strongly coupled polariton systems [2] and as the basis for intersubband devices in a system not affected by polar optical phonons [11], [12]. Ge-based devices are also opening new avenues towards better mid-IR modulators [13] and waveguides [14].

The emergence of novel materials such as graphene, transition metal dichalcogenides (TMDs), and topological insulators has expanded the palette of possibilities. Graphene, with its unique electronic properties and tunability, holds promise for IR and THz devices, offering high carrier mobility and broadband response, aspects that are crucial in many applications. The synergy between these novel materials and intersubband transitions offers rich opportunities for the development of next-generation IR and THz quantum devices. For instance, integrating graphene with semiconductor heterostructures enables the exploration of hybrid systems with enhanced functionalities like the establishment of mode-locking and frequency comb operation in quantum cascade lasers [15], [16]. Likewise, leveraging intersubband transitions in emerging materials like TMDs holds the promise of ultrathin, high-speed devices that represent attractive candidates for applications ranging from photodetection to modulators. Furthermore, topological insulators, with their robust surface states, present an exciting avenue for novel plasmonic device architectures in the IR and THz regime [17].

Plasmonics is a research area whose potential in the mid-IR and THz is still to be fully tapped. Collective plasma waves in semiconductors can give rise to interesting quantum effects manifesting themselves in peculiar optical properties [18] and optically controlled carrier dynamics [19]. Metallic microstuctures, however, remain the basis for the implementation of photonic crystals, metamaterials, cavities and antennas, which are now finding new exciting applications for the enhancement and optical control of magnetism and spintronic effects [20], [21], [22], [23]. Furthermore, they allow engineering of ultra-strong light–matter coupling with inter-Landau-level transitions [24], improved geometries of THz detectors [25] and novel waveguiding concepts for THz signals [26].

In conclusion, the perspective for infrared and terahertz quantum devices, propelled by the exploration of novel materials and intersubband transitions, is poised for significant advancements. The synergistic interplay between material science, quantum physics, and device engineering holds the key to unlocking the full potential of these emerging technologies.

As researchers delve deeper into the fundamental properties of materials and quantum phenomena, we anticipate a future where IR and THz quantum devices become indispensable tools, revolutionizing fields ranging from communications and sensing to imaging and beyond.
